# Ownership and Use of Insecticide-Treated Nets among People Living in Malaria Endemic Areas of Eastern Myanmar

**DOI:** 10.1371/journal.pone.0162292

**Published:** 2016-09-12

**Authors:** Tin Aung, Chongyi Wei, Willi McFarland, Ye Kyaw Aung, Hnin Su Su Khin

**Affiliations:** 1 Population Services International-Myanmar, Yangon, Myanmar; 2 University of California, San Francisco, San Francisco, California, United States of America; Academic Medical Centre, NETHERLANDS

## Abstract

**Background:**

Myanmar has the highest burden of malaria in the Greater Mekong. However, there is limited information on ownership and use of insecticide-treated nets (ITNs) in areas of Myanmar most severely affected by malaria. We describe ownership and use of ITNs among people in the malaria-endemic eastern parts of Myanmar and factors associated with ITN use.

**Methods:**

A cross-sectional household survey using a multi-stage cluster design was conducted in malaria-endemic townships in eastern Myanmar during the high malaria season of August to September, 2014. An effective ITN was defined as 1) a long-lasting insecticide-treated net obtained within the past three years, or 2) any net treated with insecticide within the past year.

**Results:**

In 4,679 households, the average number of ITNs per household was higher in rural compared to urban areas (0.6 vs. 0.4, p <0.001) as well as the proportion of households owning at least one ITN (27.3% vs. 15.5%, p<0.001). The proportion of households in which all members slept under an ITN was also higher in rural compared to urban areas (15.3% vs 6.9%, p<0.001). In multivariate analysis, rural households (adjusted odds ratio [aOR] 1.78, 95% CI: 1.43–2.21, p<0.001), households in which respondents knew malaria is transmitted by mosquitoes (aOR 1.35, 95% CI: 1.10–1.65, p = 0.004), and in which respondents knew malaria can be prevented by ITN use (aOR 1.86, 95% CI: 1.28–2.70, p<0.001) were more likely to have all members sleep under an ITN. Compared to the lowest socio-economic quintile, households in the richest quintile were less likely to have all members sleep under an ITN (aOR 0.47; 95% CI: 0.33–0.66, p<0.001). Households in which the main income earner was a skilled worker or a businessman were less likely to have all members sleep under an ITN (aOR, 0.70, 95% CI: 0.52–0.96, p<0.025) compared to those headed by farmers or fishermen. Households in which all children slept under an ITN were more likely to be in rural areas (aOR 1.58, 95% CI: 1.19–2.09, p = 0.002) and have a household head who knew malaria can be prevented by ITN use (aOR 2.13, 95% CI: 1.30–3.50, p = 0.003). Children were less likely to have slept under an ITN in houses headed by skilled workers or businessmen (aOR 0.50, 95% CI: 0.33–0.75, p = 0.001) or unskilled workers (aOR 0.66, 95% CI: 0.49–0.89, p = 0.006) compared to households with farmers or fishermen. Higher socio-economic level was associated with lower ITN use by children (aOR 0.56, 95% CI: 0.36–0.88, p = 0.012, highest vs. lowest quintile).

**Conclusions:**

The study found ownership of ITNs was low in Myanmar in comparison to the goal of one for every two household members. Use of ITNs was low even when present. Findings are of concern given the study areas were part of enhanced efforts to reduce artemisinin-resistant malaria. Nonetheless, groups vulnerable to malaria such as individuals in rural settings, lower socio-economic households, and workers in high mosquito exposure jobs, had higher rates of ITN ownership. Malaria knowledge was linked to effective ITN use suggesting that distribution campaigns should be complemented by behavior change communications.

## Introduction

Myanmar has the highest burden of malaria among countries of the Greater Mekong, contributing nearly three-fourths of malaria cases in the region [1]. In 2014, WHO reported nearly 300,000 confirmed malaria cases in Myanmar where *Plasmodium falciparum* infection accounted for about 70% of all cases. Nearly 60% of Myanmar’s population lives in malaria endemic areas with 34% of this population living in high and moderate risk areas [1]. Much transmission occurs in forest areas and their fringes; thus all individuals living in households located within one kilometer of forest areas are at highest risk [2]. When the house is located farther away, the risk is typically high for adult males who visit the forest for activities such as logging, mining, agriculture, and road or dam construction [2]. Within townships (Myanmar’s primary administrative unit) in malaria endemic areas, the risk is higher in rural compared with urban areas due to the proximity to forests and the engagement of rural people in forest-related jobs [2].

**Fig 1:**
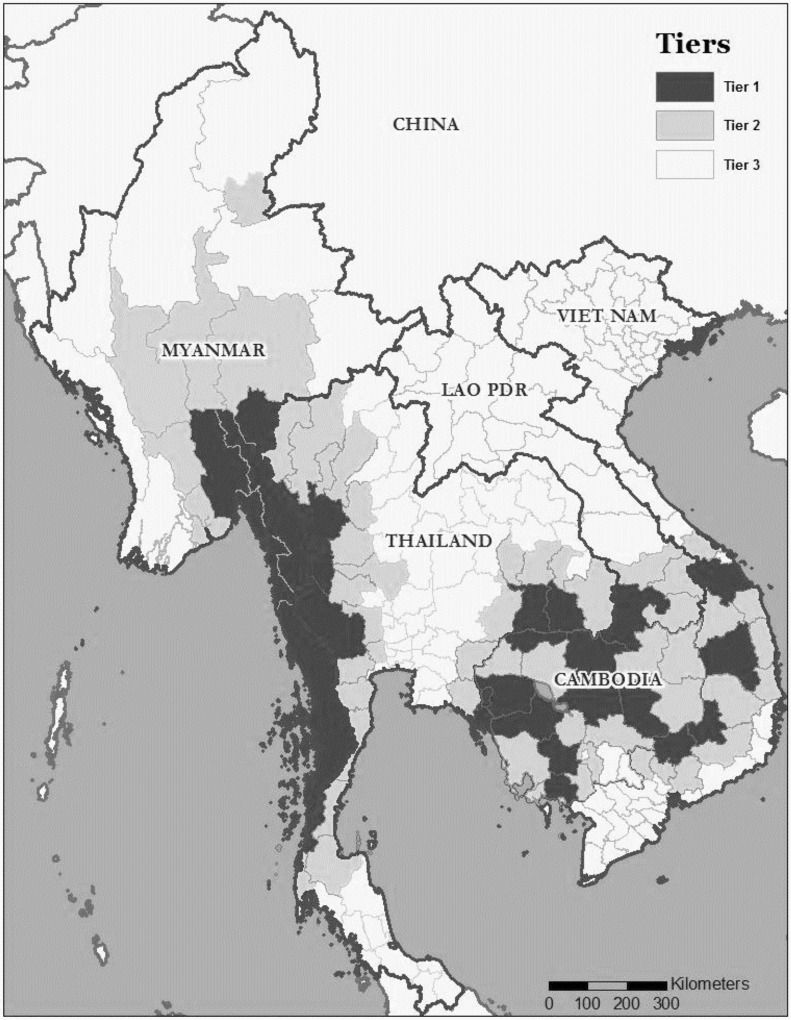
Map of the Greater Mekong sub-region, showing artemisinin resistance areas (adapted from the WHO Status Report on Artemesinin Resistance, January, 2014[9]). **Tier 1:** Areas for which there is credible evidence of artemisinin resistance, where an immediate, multifaceted response is recommended to contain or eliminate resistant parasites as quickly as possible. **Tier 2:** Areas with significant inflows of mobile and migrant populations from tier 1 areas or shared borders with tier I areas, with intensified malaria control to reduce transmission and/or limit the risk of emergence or spread of resistant parasites. **Tier 3:** *Plasmodium falciparum* endemic areas which have no evidence of artemisinin resistance and have limited contact with tier I areas, where prevention and preparedness should focus on increasing coverage with parasitological diagnostic testing, quality-assured artemisinin-based combination therapy and vector control.

Insecticide-treatment nets (ITNs), including long-lasting insecticide-treated nets (LLINs), can be effective in reducing malaria morbidity and mortality [3]. Myanmar’s National Strategic Plan for 2010–2015 envisioned the reduction of malaria morbidity and mortality by 50% partly through the goal of 80% protection of the population by ITNs in moderate and high risk villages in 284 townships [2]. The emergence of artemisinin-resistant falciparum malaria in Cambodia and Myanmar in 2011 led to a strategic decision of targeting universal (100%) coverage of ITNs in areas with credible evidence of artemisinin resistance (i.e., “Tier 1” areas, Fig 1). Bed net distribution goals for Myanmar were to achieve one ITN for every two persons [4]. From 2011 to 2014, more than seven million ITNs and LLINs were distributed in 280 townships with nearly half going to the 52 Tier 1 townships in Myanmar [5].

Although millions of ITNs and LLINs have been distributed over the past three years, only limited studies have described their ownership and use among specific groups of people in Myanmar [6, 7, 8]. No large-scale, population-based survey has documented the coverage of effective ITN ownership and use among people living in urban and rural areas to assess progress towards achieving Myanmar’s 2015 goal. Thus, the objective of our study was to fill this gap by describing effective ITN ownership and use of households in eastern Myanmar. We also sought to characterize determinants of effective ITN use for all household members and among all children under five years of age in households with such children.

## Materials and Methods

### Study area

The study area consisted of 64 malaria endemic townships located along the eastern part of Myanmar including the Tier 1 resistance containment area in the southeast panhandle (Fig 1) as well as other townships along eastern border. All townships included were either at high or moderate risk of malaria. The study area included approximately six million people. The study area was defined as the southeastern panhandle and the eastern border areas. The study sample was designed to be proportionate to size at the level of township and primary sampling unit (wards and village tracts that make up the urban and rural townships respectively); however, survey weights were needed to account for differential probability of inclusion by rural/urban strata.

### Sampling design

The population-based, cross-sectional household survey was conducted in the peak malaria transmission season of August to September in 2014. The sample was drawn using a multi-staged, cluster sampling approach. A study sampling frame included all townships in the study area, except those that were inaccessible for security reason (28 townships where about 16% of the population lived), and the two largest urban areas of Yangon and Mandalay. Within each township, urban areas were sub-divided into wards and in rural areas into village tracts. Wards and village tracts were defined as the primary sampling units or clusters in our study. On average, urban areas had about six to eight wards per township area and rural areas had about 30 to 35 village tracts per township. Rural areas were larger and also had about 70% of the population. In the first stage, we randomly selected 13 townships by probability proportionate to size. In the second stage, within each selected township we randomly selected four wards and four village tracts also by probability proportionate to size. Thus, in total 52 wards and 52 village tracts were included in the sample. In the third stage, within each selected ward or village tract, the survey team mapped and enumerated all households and selected 45 households in each cluster by systematic random sampling (i.e., a random start and interval to cover the entire ward or village tract). The estimated number of households in each ward and village tract was obtained from the ward/village leader to determine the sampling interval to select the households.

The survey team visited 5,984 selected households and a total of 4,679 household representatives were successfully interviewed. If the eligible household head was not available, the research team asked when that person would return and make a second visit within the survey team’s stay in the area (usually about 3–4 days). If the eligible person would not return within that period, the household was replaced with the nearest occupied household. For 1,185 of the 5,984 selected households (19.8%), the eligible representative was not available. For 119 (2.0%), the eligible person refused to be interviewed. These cases were replaced by the nearest occupied household.

### Recruitment procedures and measures

Interviewers explained the purpose of the study and obtained verbal informed consent from the head of the household or their spouse. In cases where neither such person was available, one of the present adult members of the household was selected to interview. The interview was conducted at participants’ homes at their time of convenience. Data were collected on paper using pre-coded structured questionnaires by face-to-face interview in the local language. Staff received three days of training on the purpose of the study, how to select a household, how to identify an eligible respondent, how to obtain informed consent, and how to administer the questionnaire.

The survey instrument included three sections. The first section recorded household characteristics such as the number of members, their ages, and a series of asset questions to assess gauge socio-economic status. The second section collected demographic characteristics of the household head, such as age, sex, education, and occupation of main income earner. The third section asked about net ownership and net use by the household members, and respondents’ knowledge of malaria transmission and prevention.

Two separate models were constructed to characterize correlates of 1) all household members sleeping under an ITN, and 2) all children under five years old in the household sleeping under an effective ITN on the night prior to the interview. To record these outcomes, interviewers asked to see each net in the household and then asked how many people slept under each and every ITN that the household owned. If the total number who slept under the combined ITN matched the number of household members, we classified the household as having all members sleeping under an ITN. Similarly, interviewers verified if all under five year olds slept under an ITN. An effective ITN was defined as either 1) a LLIN that was purchased or received within the past 3 years, or 2) any net treated with insecticide within the past 12 months. In order to identify if a net was an LLIN, the interviewer checked the brand of the net using a show card with all brands available in Myanmar. If the brand was not included on the card, the interviewer wrote down the brand name on the questionnaire and it was later recoded in the data analysis stage.

### Analysis

Data were double entered into a CSPro database system. The verified data set was converted to STATA version 13.0 for analysis. The sample design, including urban/rural weights and clustering at the primary sampling unit, was accounted for in the analysis using standard commands for complex survey designs.

We used Chi square tests to compare household characteristics, net ownership and use, and malaria knowledge between urban and rural residents. We used t-tests to compare continuous variables after confirming normal distributions. We conducted bivariate and multivariate logistic regression to identify significant correlates of the two key outcomes (i.e., if all household members slept under an ITN, and if all under 5 children of the households slept under an ITN). P-values less than 0.05 were considered significant.

### Ethics statement

Verbal informed consent was obtained from all persons participating in the study, who were the heads of households, their spouse, or another adult member of the household present at the time of the recruitment visit. After explaining the purpose and procedures of the study and making persons aware that they had the right not to participate or to withdraw from the study at any time, the interviewer signed the consent form on behalf of the respondent documenting informed consent was given. The project, including the entire consent procedures, obtained approval from the Population Services International Ethical Review Board (OHRP FWA 00009154, IRB#00006978). Confidentiality was maintained at all steps of data collection.

### Results

Table 1 shows demographic characteristics of the sample. The average household size was 4.7 members in urban areas and 4.6 in rural areas. Households were generally headed by males (80.0% urban, 82.1% rural) over the age of 50 years (54.6% urban, 47.9%). The population generally had low education levels, more so in rural areas. People living in rural areas worked more as farmers or fishermen; people in urban area tended to be skilled laborers or ran small businesses.

**Table 1:** Demographic characteristics, knowledge of malaria, ownership and use of insecticide-treated nets (ITNs) by households in urban vs. rural areas in eastern Myanmar, 2014 (N = 4,679 households).

Characteristics and indicators	Urban Average (SD) or N (%^a^)	Rural Average (SD) or N (%^a^)	p-value^b^
Total households	2,339	2,340	--
Average number of people per household	4.7	4.6	--
Households having under 5 children (n = 1,636)	764 (32.7)	872 (37.3)	<0.001
Age of household head			<0.001
18–49 years	1,062 (45.4)	1,219 (52.1)	
50 years and above	1,277 (54.6)	1,121 (47.9)	
Sex of household head			0.056
Male	1,870 (80.0)	1,922 (82.1)	
Female	469 (20.1)	418 (17.9)	
Education attained by household main income earner			<0.001
No and low education level	1,025 (43.8)	1,674 (71.5)	
Middle education level	690 (29.5)	453 (19.4)	
High education level /diploma/graduate	624 (26.7)	213 (9.1)	
Occupation of household main income earner			<0.001
Farmer/fisherman	570 (24.4)	1,243 (53.1)	
Skilled/business man	923 (39.5)	263 (11.2)	
Unskilled worker/no job	846 (36.2)	834 (5.6)	
Household respondent knew that malaria is transmitted through mosquito bites	1,667 (71.3)	1,414 (60.4)	<0.001
Household respondent knew that malaria can be prevented by sleeping under an ITN	120 (5.1)	100 (4.3)	<0.166
Average number of any net per household, mean (SD)	2.6 (1.2)	2.3 (1.1)	<0.001
Average number of effective ITNs per household, mean (SD)	0.4 (0.8)	0.6 (1.0)	<0.001
Households with at least one effective ITN	362 (15.5)	639 (27.3)	<0.001
Households in which all members slept under an effective ITN	163 (6.9)	359 (15.3)	<0.001
Households in which all children under five slept under effective ITNs	109 (14.3)	226 (25.9)	<0.001

^a^Percentages are weighted for the survey design.

^b^Chi-square test, weighted.

ITN: insecticide-treated net; SD: standard deviation.

Urban household heads had higher knowledge of the fact that malaria is transmitted through mosquito bites (71.3%) compared to rural household heads (60.4%) (Table 1). Fewer heads of households knew that sleeping under an ITN can prevent from getting malaria at 5.1% and 4.3% by urban and rural households, respectively. Regarding any net ownership, urban households owned on average 2.6 nets compared to 2.3 among rural residents. Considering only effective ITNs, rural households had 1.0 on average compared to 0.8 among urban households. Overall, 15.5% of households in urban areas and 27.3% in rural areas owned at least one effective ITN. The proportion of households in which all members slept under an effective ITN was significantly higher among the rural compared to the urban population (15.3% vs. 6.9%, respectively, p<0.001). Among households with children under five years old, the proportion in which all children under five slept under effective ITN was higher among rural households compared to urban (25.9% vs. 14.3%, respectively, p<0.001).

Bivariate and multivariate correlates of all household members sleeping under an ITN are presented in Table 2. A higher likelihood of all household members sleeping under an ITN was found in rural areas (adjusted odds ratio [aOR] 1.78, 95% CI: 1.43–2.21, p<0.001) and in households where the head knew that malaria is transmitted by mosquitoes (aOR 1.35, 95% CI: 1.10–1.65, p = 0.004) and that malaria can be prevented by using an ITN (aOR 1.86, 95% CI: 1.28–2.70, p = 0.001). Lower likelihood of ITN use was associated with the household head being a businessman or skilled worker (aOR 0.50, 95% CI: 0.33–0.75, p = 0.001) or unskilled worker (aOR 0.66, 95% CI: 0.49–0.89, p = 0.006) compared to farmer or fisherman. Compared to the lowest quintile, households with higher socio-economic status were less likely to have all members sleep under an ITN.

**Table 2:** Characteristics of households in which all members slept under an effective insecticide-treated net (ITN), eastern Myanmar, 2014 (N = 4,679 households).

Characteristics	All members slept under effective ITN	Unadjusted OR (95% CI)	p-value^a^	Adjusted OR (95% CI)	p-value^b^
Residency					
Urban	163 (31.2%)	Reference	--	Reference	--
Rural	359 (68.8%)	2.42 (1.99, 2.94)	<0.001	1.78 (1.43, 2.21)	<0.001
Sex of household head					
Female	108 (20.7%)	Reference	--	Reference	--
Male	414 (79.3%)	0.88 (0.71, 1.11)	0.284	0.82 (0.65, 1.04)	0.097
Age of household head					
18–49 years	284 (54.4%)	Reference	--	Reference	--
50 years and above	238 (45.6%)	0.77 (0.65, 0.93)	0.006	0.82 (0.68, 1.00)	0.048
Education attained by household main income earner					
No or low education	350 (67.1%)	Reference	--	Reference	--
Middle education	106 (20.3%)	0.69 (0.55, 0.86)	<0.001	0.84 (0.66, 1.07)	0.161
High/diploma/graduate	66 (12.6%)	0.57 (0.44, 0.76)	<0.001	0.92 (0.68, 1.25)	0.593
Occupation of household main income earner					
Farmer/fisherman	241(46.2%)	Reference	--	Reference	--
Skilled/business man	77 (14.8%)	0.45 (0.35–0.59)	<0.001	0.70 (0.52–0.96)	0.025
Unskilled worker	204 (39.1%)	0.90 (0.74–1.10)	0.309	0.89 (0.71–1.12)	0.317
Household head socio-economic status					
Level 1 (poorest)	152 (29.1%)	Reference	--	Reference	--
Level 2	145 (27.8%)	0.95 (0.74, 1.22)	0.709	0.93 (0.71, 1.22)	0.613
Level 3	105 (20.1%)	0.66 (0.51, 0.87)	0.003	0.71 (0.53, 0.94)	0.015
Level 4	64 (12.3%)	0.37 (0.27, 0.51)	<0.001	0.47 (0.34, 0.66)	<0.001
Level 5 (richest)	56 (10.7%)	0.33 (0.25, 0.46)	<0.001	0.47 (0.34, 0.66)	<0.001
Knew malaria is transmitted through mosquito bites	354 (67.8%)	1.10 (0.91, 1.34)	0.314	1.35 (1.10, 1.65)	0.004
Knew malaria can be prevent by sleeping under ITNs	39 (7.47%)	1.77 (1.24, 2.54)	0.002	1.86 (1.28, 2.70)	0.001

^a^Bivariate logisitic regression analysis.

^b^multivariate logistic regression analysis.

ITN: insecticide-treated net; OR: odds ratio; CI: confidence interval.

For the sub-sample of households with children under five years old, correlates of all children using ITNs are presented in Table 3. All children were less likely to have slept under an ITN in households headed by skilled workers or business men (aOR 0.50, 95% CI: 0.33–0.75, p = 0.001) or headed by unskilled workers (aOR 0.66, 95% CI: 0.49–0.89, p = 0.006) compared to households headed by farmers or fishermen. Higher socio-economic level was also associated with lower likelihood of all children sleeping under an ITN (aOR 0.56, 95% CI: 0.36–0.88, p = 0.012 for the highest quintile vs. lowest).

**Table 3:** Characteristics of households in which all children under 5 years old slept under an effective insecticide-treated net (ITN), eastern Myanmar, 2014 (N = 1,636).

Characteristics	All members slept under effective ITN	Unadjusted OR (95% CI)	p-value^a^	Adjusted OR (95% CI)	p-value^b^
Residency					
Urban	109 (32.5%)	Reference	--	Reference	--
Rural	226 (67.5%)	2.10 (1.63, 2.70)	<0.001	1.58 (1.19, 2.09)	0.002
Sex of household head					
Female	39 (11.6%)	Reference	--	Reference	--
Male	296 (88.4%)	1.39 (0.96, 2.00)	0.08	1.33 (0.90, 1.95)	0.149
Age of household head					
18–49 years	207 (61.8%)	Reference	--	Reference	--
50 years and above	128 (38.2%)	0.90 (0.71, 1.15)	0.414	1.08 (0.83, 1.41)	0.561
Education attained by household main income earner					
No or low education	217 (64.8%)	Reference	--	Reference	--
Middle education	80 (23.9%)	0.88 (0.66, 1.17)	0.386	1.08 (0.79, 1.48)	0.615
High/diploma/graduate	38 (11.3%)	0.52 (0.36, 0.75)	0.001	0.74 (0.49, 1.11)	0.148
Occupation of household main income earner					
Farmer/fisherman	164 (49.0%)	Reference	--	Reference	--
Skilled/business man	43 (12.8%)	0.37 (0.26–0.54)	<0.001	0.50 (0.33–0.75)	0.001
Unskilled worker	128 (38.2%)	0.72 (0.55–0.94)	0.015	0.66 (0.49–0.89)	0.006
Household head socio-economic status					
Level 1 (poorest)	95 (28.4%)	Reference	--	Reference	--
Level 2	79 (23.6%)	0.75 (0.53, 1.06)	0.104	0.62 (0.42, 0.90)	0.012
Level 3	72 (21.5%)	0.71 (0.50, 1.01)	0.055	0.64 (0.44, 0.94)	0.023
Level 4	43 (12.8%)	0.40 (0.27, 0.59)	<0.001	0.44 (0.28, 0.67)	<0.001
Level 5 (richest)	46 (13.7%)	0.45 (0.30, 0.67)	<0.001	0.56 (0.36, 0.88)	0.012
Knew malaria is transmitted through mosquito bites	226 (67.5%)	1.05 (0.81, 1.36)	0.696	1.28 (0.98, 1.69)	0.074
Knew malaria can be prevent by sleeping under ITNs	28 (8.36%)	2.11 (1.31, 3.38)	0.002	2.13 (1.30, 3.50)	0.003

^a^Bivariate logisitic regression analysis.

^b^multivariate logistic regression analysis.

ITN: insecticide-treated net; OR: odds ratio; CI: confidence interval.

## Discussion

This study found low ownership of effective ITNs in both urban and rural areas of eastern Myanmar. With fewer than one in six urban households and one in four rural households owning ITNs, estimates fell far short of the 100% coverage targeted for Tier 1 areas by 2015. Use defined by all members of the household sleeping under an ITN was even lower–on 6.9% in urban areas and 15.3% in rural areas.

This study also found far fewer effective ITN per capital than recommended by the Myanmar National Malaria Control Program guidelines, indicating low distribution and/or retention of nets. The guidelines recommend there be one ITN distributed for every two persons. Our survey counted about one ITN per 10 persons. Findings are disappointing given that a 2013 study in the Kachin special region found one LLIN for every two and half people and over three-quarters of people sleeping under an effective ITN the preceding night [6]. One possible reason is that the survey was implemented after repeated rounds of LLIN distribution campaigns within a smaller geographic area and population. High ITN ownership and use was also found in two other evaluation studies carried out in well-defined populations and in Tier 1 areas. One was among 350 migrant workers finding 82.3% ITN ownership and 53.1% ITN use [7]. The other was among 800 households assessing the effectiveness of a behavior change communication program in which 75.1% of households owned at least one ITN or LLIN for every two persons [8]. These evaluations also shortly followed mass distribution efforts (i.e., within six to 12 months). Sustain high coverage and use of ITNs are essential to the success of malaria control programs, requiring monitoring ownership and use over time. Notably, the present study was independent of distribution campaigns and also carried out in a larger population-based sample covering eight states and divisions with six million people.

We also found several strong correlates of household ITN use, including living in a rural area, occupation of farmer or fisherman, belonging to poorest socio-economic quintile, and respondents having malaria-specific knowledge (i.e., knowing how malaria is transmitted and that it can be prevented). Higher ITN use among rural households could be due to the specific targeting of distribution programs. Likewise, the higher proportion of poorer households using ITN could be explained by the priority given to ensuring equity during net distribution campaigns [4]. The finding of farmer or fisherman more likely to use ITN could be due to the perception of their jobs being higher risk of getting malaria compared with other groups such as unskilled workers, skilled workers, or businessmen. The perception is consistent with the context of risk in Myanmar context where agriculture and forest related jobs are of higher risk than among skilled workers and businessmen [2]. Similarly, correct perception of risk is likely related to malaria specific knowledge, such as transmission by mosquitoes, as found in our study. Having knowledge of malaria prevention was also associated with ITN use in our study. These findings support the potential effectiveness of malaria behavior change communication campaigns targeting persons in high-risk occupations and areas.

Encouragingly, we found that the proportion of households with children under five years old sleeping under an ITN was substantially higher than of that of all household members. This was true for both rural and urban households. The prioritization may be due to the perception that children are more vulnerable to malaria mortality. However, the reasons for why children were more likely to sleep under ITN than adults were not probed. Data are also needed on ITN use by mobile men when away from home and in forest areas where their acquisition risk is high. Future studies using qualitative techniques should be conducted in order to explore household arrangements, reasons for use of INT within household members, and strategies for use among mobile men in forest-related jobs.

There are a number of additional potential limitations to this study. First, an effective ITN was defined as one obtained within the past three years. However, interviewers were unable to identify the actual manufacturing dates, instead relying on household heads reporting when the net was received, with inherent recall bias. Second, we asked how many people slept under each and every ITN that the household owned, and if the total number slept under all the combined ITN matched with the number of household members present, we considered it as all members slept under ITN. We did not take into account those people who might be visiting and slept at home on the night before. Third, a total of 1,304 initially selected households had been replaced potentially introducing bias. While the direction of possible bias due to these replacements is not known, such replacements were more likely to affect the data in rural areas as the survey was carried out in the rainy season when farmers are far away from their houses and without spare members to respond.

## Conclusions

Study findings highlight the huge gap to achieve the 100% coverage of ITN or LLIN that is the target for all people living in these malaria endemic areas. Ownership and use of an effective ITNs are still low in malaria-endemic and artemisinin-resistant areas of Myanmar. However, it is encouraging that efforts are reaching vulnerable populations such as rural and poorer people. Scaling up efforts can lead to increased ITN coverage which may contribute but not necessarily reduce malaria morbidity and mortality. Our finding that knowledge is strongly linked to effective ITN use suggests that net distribution campaigns should be complemented by behavior change communication emphasizing that malaria is transmitted by mosquito bites and it can be prevented by sleeping under ITN. Qualitative studies are needed to explore the household decision-making and arrangements for use of ITN among household members as well as ITN use by mobile, male populations.
